# Essay Read before the Late Dental Convention at Niagara Falls

**Published:** 1859-10

**Authors:** Jas. Taylor


### ARTICLE VII.

*Essay Read before the late Dental Convention at Niagara Falls.* By Prof. JAS. TAYLOR, M. D. Reported for the American Journal of Dental Science.

"When, and under what circumstances should teeth be extracted, to secure a correct development of the permanent teeth?"

MR. PRESIDENT:—From the manner in which this question is presented, one or two special thoughts are suggested, which, although not in direct answer to the subject under discussion, yet, as they may give a profitable turn to the manner in which this question may be considered, I will dwell upon for a moment. The first thought is this: can we by the removal of the deciduous organs—for these are the ones almost necessarily implied as to be removed—in any way effect the development of the permanent organs? Is there not a law of the economy which removes the one and develops the other, which presides over all that change which is carried on during this interesting and important period of dentition?

The natural and uninterrupted operations of the economy should be sufficient, and generally are, to effect all the changes which take place in the system, yet there are cases where from disease and other causes, the natural function is disturbed. Still the question comes up, will the removal of a deciduous tooth at any period, effect the development of the tooth of replacement? can the presence of the former, by pressure or other means, prevent the full development of the latter? We know that the organs engaged in the assimilation and production of the one, are independent of those employed for the destruction of the other. The idea has been entertained, and perhaps very justly, that the development of the one was necessary to the removal of the other. That without the pressure thus induced, absorption of the fang would not take place, or at least that the growth of the one was necessary to carry forward the absorbing or destroying body prepared by an all-wise Providence for the destruction of the roots of the other. We take the twenty teeth of replacement, and do we ever find them malformed or diminished in size by the long retention of the deciduous teeth? One of our most celebrated dentists, on the examination of the mouth of a young lady of seventeen or eighteen, which presented an upper cuspid not yet shed, and no appearance of the permanent organ, was asked if he would remove the temporary. His answer was, not now, but at fifteen he would have done so. The idea was this, the permanent organ had acquired a position somewhere in the maxillary arch, which it would now hardly leave; whereas, if the way had been made clear at fifteen, it might have come in place. The question might be asked still with propriety, was not that hidden cuspid fully developed? We believe that such have been found usually fully developed.

The malformed teeth which are often found in the teeth of replacement, are not usually supposed to be thus effected by the confined condition of the organ, when in a formative state. We have seen the *dens sapientiœ* when full development appeared, defective for want of room, and often recommend the removal of a defective molar, hoping thereby to secure a more perfectly developed and enlarged organ to take the place of the last. Observation appears to confirm this supposition, yet the evidence is hardly of a demonstrable kind, for we cannot tell what the organ might not have been if left alone to work its more slow eruption.

Another thought is suggested to my mind, and deserves a passing consideration. The deciduous organs become involved in disease, and will this disease prevent the development of the organs of replacement?

We do not deny that alveolar abscess at the apex of the root or roots of the deciduous teeth, may not so involve the surrounding parts in the disease, as to destroy the sac in which the permanent germ is placed, and even the germ itself. Yet this does not often occur, and, perhaps, partly because the crown of the replacing tooth is most usually developed before this takes place, and hence we so often see shortly after the formation of an abscess, and the removal of the tooth which caused it, a new and beautiful tooth take its place. Not as often do we find the enamel of the new tooth eroded or injured, as we would suppose. The earlier in life these abscesses occur the more apt they are to injuriously effect the permanent organ, because the deposition of enamel and the formation of dentine has not been perfected in the new organ.

But one other thought in this connection, and the most important one is this: To what extent can disease at all, local or constitutional, effect the development of the dental organs? The earliest authors reasoning from analogy, very justly infer that general constitutional disease must necessarily impair the integrity of the teeth, and hence to the present time there is a kind of vague idea that organs so intimately connected with the general organism, must suffer with it whenever involved in general disease. Even the mother's health is supposed to exert an influence on the teeth of her child. All the researches made in physiology point to the fact that perfect development can only take place in perfect health, yet to what extent, the size and form of any organ may be affected, is a matter of much doubt and speculation. With regard to the dental organs, we believe that the general supposition is, that the effect is more constitutional than physical. Are we, however, sure that osseous development which marks the peculiar physical characteristics of the different races of mankind may not be more or less dependent on the kind of nutrition supplied, can climate and locality alone be the cause of all the varied physical characteristics presented to our view. There are marked hereditary tendencies we admit; this is often seen in the form of the maxillary arches, and often present one of the greatest obstacles to the treatment of irregularity of the teeth. Can we expect by change of diet and proper treatment of irregularity in a few generations, to overcome this hereditary tendency? Will like produce its like, according to the laws of germinal force, or can that germinal force which, for instance, gives a protruding chin, be so changed by diet and art that in a few generations it will be overcome? Can the profession give instances where the parent's teeth have been hereditarily corroded and thrown out of position; and when this has been remedied by art; and where the like tendency in the children's teeth does not exist? If this tendency appears to be overcome, can it be attributed to the want of the like hereditary tendency in one of the parents? In all animated nature there appears to be a germinal power, (if we may so call it,) a power which sets in motion the springs of life, and which controls to a certain extent, the future of that life. Still, we do see, in the vegetable kingdom, and we believe in the animal also, wondrous changes wrought by the nutrition supplied, and the trimming and pruning sometimes given. It is known that the basis of all bone and dentine is phosphate of lime, and that certain articles of diet afford a far richer supply of these elements than others. Much attention of late has been given by the medical profession to phosphate preparations for the cure of that great scourge of our race, consumption. Why, let me ask, not go back to the earliest germinations of life, and say to the mother, the perfect development of your child and that stamina which must sustain it through life, begins with you, and then to the child, your vigor of constitution—your manly form, and even the perfection of these ornaments of the mouth, which aid your intercourse with the world, and which commences that function of digestion on which all the assimilated powers of your system depend, now depend on your mode of living. We fear, that as a nation we soon shall have the very unenviable reputation of worthless. We are certainly the most indiscriminate feeders in the world. An all-wise Providence has given us a land flowing with "milk and honey," and indeed almost every other good thing. Are we, with all our boasted intelligence, using these mercies to the great advance of human life? As a profession, we are perhaps doing more than any other nation to artistically and surgically meet the wants created by dental disease. Would it not be well to, as far as possible, arrest that disease itself? We may take a stunted, feeble-looking shrub, and by transferring it to good soil, and proper culture, develop, as it were, a new life; so that its after-growth shall forever hide its feeble beginning. Are these vegetable creations around us of more value than the dear ones entrusted to our charge? The law which govern the development of the dental organs appear to be different from that which govern true osseous-structure, and it is certainly, to a certain extent, independent of it.

We are not certain but that crowded dentures are the result more of imperfect osseous development, than dental. The very alveòlar processes, which are not dental in their structure, appear to want full development to meet the demands of the dental system. We but seldom find a crowded denture when the maxillary arches are full formed. The teeth are moulded for large jaws, but the health which govern the full development of the general frame, have been so long violated and neglected, that the frame is necessarily too small. The teeth are very properly considered the most unerring indexes by which to distinguish the inherent constitutional tendency of the system. In their physical characteristics they change less. By change of diet, location, &c., the general physical traits may almost lose their original impress.

There can be no doubt but that the constitution of the teeth is more or less effected by the kind of nutrition supplied during their formative state. We are not sure but that the refinements of civilization, while pampering to the taste and eye of our race, have not stripped our food of the very elements we most need. The aborigines of our land, and even the hardy pioneers of our race, possessed, as a general rule, larger frames, and more enduring constitutions than we do, that their dental organs were better, and particularly the former, there can be no doubt. We are not speaking of what they are now, for they have changed, and their alveolar arches will not fail to present such specimens of teeth as are generally found in the sepultures of their dead. In all the bounties thrown around us for our support, certain elements exist in proportions about such as are demanded for the elaboration and reparation of our systems. All our aliment is supposed to contain one of three important principles necessary to animal life. These are the calorifacient or heat sustaining principles. This is found in most vegetable matter and in sugar. The nutrient principle, which is abundantly found in animal food, particularly the lean thereof, in milk, eggs, potatoes, wheat, rye, &c. The inorganic elements from which are derived those earthy constituents, which build up the frame work of the system, and hence good bone and teeth, are to be found most abundant in eggs, milk, animal food, and particularly in wheat, rye, oats, potatoes, &c. Bread is said to be the "staff of life." Let us see how much of the real staff is taken from the wheat before we now get our bread. Prof. Johnson in his analysis gives us the following:

| | |
|---|---|
| In 1,000 lbs. of whole grain, there were muscle material, | 156 lbs. |
| Of the fat principle, | 25 " |
| Of the inorganic elements for bone, &c., | 170 " |
| 1,000 lbs. fine flour contained muscle material, | 130 lbs. |
| Fat principle, | 20 " |
| Bone material, | 60 " |
| 1,000 lbs. of bran contained muscle material, none. | |
| Fat principle, | 60 lbs. |
| Bone material, | 700 " |

What is the difference here? It is this, that the refinements of civilization have so refined, at least this one article of our food that it is hardly fit for the elaboration of bone material or for our dental organs. We have taken from the wheat more than three-fourths of those elements needed in this operation. We take our corn, rye, and many other articles of our food, and the same thing is done to a considerable extent. The Scotch, who generally present us with good specimens of teeth, do not thus serve their oatmeal, which makes, to some extent, a national article of food. But this subject, however interesting and instructive, would lead me from the question proposed for consideration. I have incidentally alluded to it because it appears to lie at the foundation of perfect dental development.

I presume, Mr. President, the aim of the question before us is, "When, and under what circumstances, should teeth be extracted to secure a regular and symmetrical denture? or, if you please, to prevent irregularity." This would appear to be the natural question in connection with the second subject presented in the programme.

Delabarre considers the eruption of the teeth as a natural parturient effort, and as such should be left as much to the natural efforts of the economy as possible. I believe that parents, and often dentists, inflict more injury than benefit by untimely interference. That there are numerous cases, however, which require aid at our hands, cannot be doubted, and there is no subject in the whole range of dental practice more difficult to definitely define than this; there is scarcely a sensible case which can be presented which might not be so effected by circumstances, so that the usual practice should not be varied.

The irregular manner in which the temporary teeth are shed, and the permanent erupted, give continued cause for change in practice; yet there are certain rules that should be, perhaps, never departed from.

Let us look at the conditions of these organs, the deciduous and permanent. Just before the loss of the one and the eruption of the other commences. Their relative position at this time is of vast importance in the proper study of this subject. "The position generally given to the germ of the permanent organs with reference to the deciduous, before the eruption of the former and the loss of the latter, is at the age of three or four years, as follows: We give Delabarre's description of their position in the inferior maxillæ." "Near the symphysis perpendicularly and deeply underneath the temporary incisors, whose roots at this period are incomplete, we shall find the crowns of the central incisors of replacement. The lateral incisors are behind, situate obliquely from the outside of the inside of the jaw, and cover a third of the posterior labial part of the central incisor, and about half of the cuspid or canine. This last is situated lower, more deeply and farther on them than the one just mentioned, and lies obliquely on the medial line, so that the three teeth form a triangular group, resembling that which we make with our three fingers in touching an angular index, thus bringing the middle one above the ranks. At a little distance from the cuspid, and immediately between the roots of the temporary molars, we find the bicuspids enveloped in their membranes, an appendage of which is carried backward to reach the gum, so that the bicuspids appear to be covered by the crowns of the milk molars.

Each kind of teeth thus ossified can be distinguished by their points; the incisors may be known by three small tubercles that surmount the cutting edge of each, the cuspids by one alone, which forms the summit of a cone. The bicuspids by two ossified points, and the molars by four.

As ossification gradually covers the pulp or dental ganglion, their points are united and form a sort of chapeau, that may be easily detached, though intimately united to it, but by vessels of so delicate a nature that the least effort breaks them. We may understand the order in which each series of teeth is placed, by taking for a comparison the ellipsis described by the gums; then drawing, in our imagination, three horizontal lines upon it, which shall be parallel to it, and pass over the summits of the teeth in ossification; at four years after birth we shall find the central incisors upon the level of the second molars, the lateral incisors upon that of the first molars and the bicuspids; while the cuspids occupy the lowest line; this is worthy of our special notice. These two classes of teeth, the temporary and permanent, are, therefore, developed at nearly the same period; but it is only when the first adorns the mouth of the child that nature directs all her efforts to those that are to succeed them. This attentive anatomical observation has not only shown us that the crowns of the teeth are formed at a very early period, but also how, from the time of their manifestation, they take their transverse dimensions, such as they should have through the whole course of life, and that the portion of the circle which the six anteriors occupy in the jaw, is at first of very limited extent, in order to allow them to be arranged at the side of each other. Nature has so arranged or disposed of them, that each covers about one-third of another, which is precisely such an arrangement as we observe in the tiles upon houses. This disposition gives them such an obliquity that supposed lines traversing their greatest diameter would cut the maxillary bone obliquely. This arrangement of the teeth is found in all jaws, it is symmetrical, it is constant and may be observed in all young subjects, it is a law from which nature never can and never does depart, it is, in short, an ingenious means employed by her to supply the want of space.

Many writers have either not appreciated the importance of these imbrications, or have not sufficiently studied on different subjects what they have pleased to consider as irregularities resulting from a malformation of the jaws; many mistakes in practice have resulted from this error.''

We confine ourself at present to the arrangement of the teeth in the inferior maxillas. The change in the position of these organs in the superior is relatively not great, and hardly requires separate description for the present discussion. We pass over also a few remarks in relation to development of the two sets of teeth, suggested by the remarks of Delabarre, and come at once to consider the consequences resulting from the too early loss of the deciduous organs. We must bear in mind the arrangement which has been given. The central incisors being on a horizontal line with the second molars. These are of course the permanent organs of which we are speaking. We have next a horizontal line a little lower down, and on this line we have the lateral incisors, the first molars and bicuspids, and still a little lower we have the cuspidati, and these, as Delabarre remarks, lie obliquely on the medial line.

With this view of the organs, before their eruption, and also before the loss of any of the deciduous set, we are prepared first to ask what purpose does the deciduous organs subserve, independent of their use in the mastication of food, or does their presence have any effect in controlling the position and direction of the permanent teeth. We think there can be no doubt of this, first because they are necessary to the continued expansion of the alveolar arch, and second, when lost they disturb that arrangement of the permanent germs which an all wise Providence has selected as the best. Their too early removal may destroy their passage for the eruption of the permanent, which the gradual destruction of the roots of the deciduous gives, or as Delabarre suggests, the *iter dentium* which appears to be a natural guide for the passage of the permanent organ may be torn away and the tooth left to seek its own exit through the gum or even remain imbedded therein, and before the crown of the permanent has ossified, and the deposition of enamel has taken place, the sac and germ may be entirely destroyed. The roots of the deciduous organs appear to be necessary to keep somewhat separate the germs of the permanent, and hence, their early loss occasions the approximation of two or more of the permanent, and their alveolar processes being arranged according to this new position, the most difficult cases of irregularity occur. The new alveolar border, supplied for the reception of the permanent organs, cannot take that position described, when the roots of the deciduous teeth have been removed before the advance which has been made by this operation of the economy demands it.

In this whole operation of first and second dentition, there is evidently a co-operative dependence, (if we may be allowed the expression,) a mutual co-laboring of each function, and this can be seen as early as the fourteenth week of utero-gestation, when the cavities of reserve are thrown off from the inner opercula of the follicles formed for the reception of the deciduous germs.

We will now more particularly notice some of the bad effects of the too early removal of the temporary teeth and also explain what we regard as the indications for their removal. The first and most common case that presents itself is the removal of the lateral incisors to give more space for the permanent central, which appears to be forced within the circle. For a time, the removal of this tooth may appear to have the desired effect, yet the mischief lies buried beneath the gum, for the root of the lateral incisor separates the germ of the lateral of the permanent set from the central, and being removed, the germ of the lateral of the permanent set, which is on the line a little below, drops forwards as it advances into the space thus made and takes the very position occupied by the deciduous one. If now the tardy eruption of the cuspidati should give room for the lateral incisor of the permanent set, it will be at the expense of all room for the former, and as the bicuspid usually come in place first, there is no place but within or without the circle for the cuspidati. We have here the natural process by which is obtained what is vulgarly called a snag tooth.

The absorption of the roots of the deciduous teeth is generally accomplished just in accordance with the wants of the permanent organs, and forms the most natural opening for them. It should, however, be borne in mind that the cavity of reserve which contains the germ for the teeth of replacement, is attached to the inner lid of the follicle, and that from this position the permanent organ would most naturally be drawn to seek an opening through the gum, a little pointing inward; this, with the incisors, is especially the case, and hence, too, that carneous body interposed for the destruction of the roots of the deciduous teeth, and which appear to be carried forward and pressed to the root by the advancing tooth, is held rather against the inner portion of the fang, and this is removed first. We thus find the outer half of the deciduous root sometimes almost perfect, while the inner is gone, this may hold the tooth apparently quite firm, and would appear to say, I am not ready to give up my position yet. But if we examine the mouth we will find on the edge of the gum, at the lingual neck of the tooth, a bulging up, showing that the permanent tooth is coming through. We now have here an indication for extraction. We have selected this starting point of second dentition on which to predicate the most of our remarks, for at this period, perhaps, more than at any other, the solicitude of the parent is most observable, and at this period are we more importuned than at any other to commence a practice which would certainly end in increased difficulty.

Parents see a little tooth soon to be lost any how, just in the way of the new central incisor, and by comparing the space and the size of the tooth, feel sure that more room must be had, and either out with the tooth, or go to the dentist and insist on having it removed. Unfortunately, this request is too often complied with.

It may, however, be asked, are there no conditions rendering the extraction of the deciduous lateral incisors necessary to give room for the permanent central? We believe not, at least we should wait until the period for their natural shedding before we resort to such treatment, we will first see what nature would effect for herself, and give her as much time for this as the nature of the case demands. In forming our diagnosis, as it regards the extraction of these teeth, we cannot be so much governed by the age of the child, as the period when the first teeth were shed, and also other conditions which present themselves. Say a child is presented that has just erupted the central incisors, and it is eight, or even nine years old; well, because our books tell us that the lateral are shed from seven to nine, it would be no excuse for their removal. We should recollect that the first being tardy, the other may be alike backward in making their appearance. This will generally be the case, so the question should not be how old is the child, but how long since such and such teeth were erupted. We have no doubt that much malpractice results from this oversight.

Decay of the deciduous molars often takes place before the eruption of the permanent incisors, and their occasional loss thus early in life has pointed out a mode of practice far preferable to the too early removal of lateral incisors, or even cuspids of the deciduous set.

To illustrate this, we present one of numerous cases of a like character.

Miss M., between seven and eight, whose central incisors (inferior) have made their appearance fully developed, but the laterals were removed to give them room. On one side, the deciduous cuspid and central permanent incisors stand almost touching each other—on the other side the space is about two-thirds of the room occupied by the lateral incisors removed. This is in consequence of the removal of a deciduous molar on this side, a year or two previous, which had been aching. Here the space has been kept up by the removal of the molar, but increased size of the alveolar border has been lost by the loss of the lateral incisors.

If, therefore, the case absolutely demands the loss of a tooth at all, we prefer to remove one of the molars. If the question were asked, which one I would remove, we would answer, that which was giving pain or trouble, or that which, by partial looseness, showed symptoms of being first shed. This practice is not likely to produce any special irregularity of the permanent bicuspids. If such is the result, it is far better here than in the front teeth, and if so, the loss of one would likely be of service to the preservation of the denture. Should the first permanent molar be decayed, and which is very likely, its loss would repair the regularity of the denture. We would, however, not advise the injudicious extraction of even any of these teeth to relieve a crowded condition of the incisors. We prefer waiting until these are well erupted, and it is evident they cannot make room for themselves. That practice which merely takes cognizance of one or two present and palpable difficulties, having no reference to collateral effects which must flow from it, is not much, if any, short of quackery.

Mr. President, the immense field of cases which lie before us in considering this subject, we leave for more accurate description by gentlemen well qualified to do them ample justice.

### EUROPEAN CORRESPONDENCE.

